# Exploiting Cancer Dormancy Signaling Mechanisms in Epithelial Ovarian Cancer Through Spheroid and Organoid Analysis

**DOI:** 10.3390/cells14020133

**Published:** 2025-01-17

**Authors:** Emily J. Tomas, Yudith Ramos Valdes, Jennifer Davis, Bart Kolendowski, Adrian Buensuceso, Gabriel E. DiMattia, Trevor G. Shepherd

**Affiliations:** 1 The Mary and John Knight Translational Ovarian Cancer Research Unit, Verspeeten Family Cancer Centre, London, ON N6A 5W9, Canada; 2 Department of Anatomy & Cell Biology, Western University, London, ON N6A 5C1, Canada; 3 Department of Oncology, Western University, London, ON N6A 5C1, Canada; 4 Department of Biochemistry, Western University, London, ON N6A 5C1, Canada; 5 Department of Obstetrics & Gynaecology, Western University, London, ON N6A 5C1, Canada

**Keywords:** epithelial ovarian cancer, high-grade serous, spheroids, organoids, cancer dormancy, transcriptome, G2/M checkpoint pathway

## Abstract

Epithelial ovarian cancer (EOC) exhibits a unique mode of metastasis, involving spheroid formation in the peritoneum. Our research on EOC spheroid cell biology has provided valuable insights into the signaling plasticity associated with metastasis. We speculate that EOC cells modify their biology between tumour and spheroid states during cancer dormancy, although the specific mechanisms underlying this transition remain unknown. Here, we present novel findings from direct comparisons between cultured EOC spheroids and organoids. Our results indicated that AMP-activated protein kinase (AMPK) activity was significantly upregulated and protein kinase B (Akt) was downregulated in EOC spheroids compared to organoids, suggesting a clear differential phenotype. Through RNA sequencing analysis, we further supported these phenotypic differences and highlighted the significance of cell cycle regulation in organoids. By inhibiting the G2/M checkpoint via kinase inhibitors, we confirmed that this pathway is essential for organoids. Interestingly, our results suggest that specifically targeting aurora kinase A (AURKA) may represent a promising therapeutic strategy since our cells were equally sensitive to Alisertib treatment as both spheroids and organoids. Our findings emphasize the importance of studying cellular adaptations of EOC cells, as there may be different therapeutic targets depending on the step of EOC disease progression.

## 1. Introduction

Epithelial ovarian cancer (EOC) is one of the most lethal forms of gynaecological malignancy, with a survival rate of less than 30% for late-stage presentation, and due to the recurrence of chemotherapy-resistant disease [1]. The most common type of EOC is high-grade serous ovarian cancer (HGSOC), which is often diagnosed at an advanced stage [2,3]. HGSOC originates from the fallopian tube epithelium, where cells acquire an inactivating *TP53* mutation, resulting in further genomic instability and increased invasiveness [4,5]. This leads to the formation of serous tubal intraepithelial carcinoma (STIC) lesions that can seed on the ovary for tumour formation [6]. These malignant tumour cells can detach and aggregate into heterogeneous clusters known as spheroids within the peritoneal cavity [7]. As these spheroids transit within the peritoneal fluid, or malignant ascites, they can adhere to the mesothelial surface and form secondary tumours. Numerous studies have shown that cells undergo changes in their pathobiology as they adapt from a tumour state to a spheroid state [8,9,10,11]. This process can be described as cancer dormancy, which explains the clinical problem of cancer recurrence from minimal underlying disease that has evaded chemotherapy [12,13]. Nonetheless, it is currently not well understood in the context of EOC metastasis.

Researchers have used three-dimensional (3D) in vitro culture model systems to investigate the specific cellular and molecular biology of EOC cells during disease progression. Spheroids have been extensively studied using suspension cultures, with Ultra-Low Attachment (ULA) plates and hanging droplet methods, to understand the mechanisms of cell survival [14]. This includes the activation of intracellular signaling pathways that induce cancer dormancy, such as cellular quiescence, cell adhesion, epithelial–mesenchymal transition (EMT), stem-like phenotype, bioenergetic stress response, and autophagy [13,14,15,16,17,18,19,20,21]. As cells transition from an adherent to spheroid culture, AMP-activated protein kinase (AMPK) activity is induced in response to cell bioenergetic stress, and protein kinase B (Akt) activity is reduced to decrease cell proliferation [18,22,23,24,25]. The process of cancer dormancy can be inhibited by targeting these specific proteins in HGSOC cells, leading to cell death within spheroids. Thus, researchers focus on discovering novel targeted agents to effectively eradicate dormant residual disease that may be evading chemotherapy-induced killing.

Several advancements have been made in 3D culture model systems, which have been integrated into current EOC studies. One such model is organoids which is very effective in replicating tumour-like structures [26,27,28]. Using an extracellular matrix (ECM) reagent, cells from any patient-derived sample (i.e., ascites fluid or dissociated tumours) can proliferate into a 3D structure that reproduces the cell–cell and cell–matrix interactions observed in vivo. With respect to EOC, various groups have made significant contributions to the development of patient-derived organoids (PDOs) and validated their ability to reproduce the patient-specific characteristics of original solid tumours [28,29,30,31,32]. As a result, PDOs have been harnessed for high-throughput drug testing of chemotherapy and many targeted agents, as well as for investigating potential mechanisms of platinum and/or Poly ADP-ribose polymerase (PARP) inhibitor resistance in patients [32,33,34,35,36,37]. Consequently, it is critical to incorporate 3D organoids into current research to comprehend the biology of HGSOC tumours and distinguish it from spheroid biology.

In this study, we compared the spheroid model system to the organoid model system using several new patient ascites-derived HGSOC cell lines to explore the underlying cellular pathobiology during disease progression. Our results indicate that EOC cells have differential expression patterns between spheroids and organoids, which validates our previous studies comparing adherent to spheroid cultures. We observed altered AMPK activity and Akt signaling when comparing adherent, spheroid and organoid cultures. However, spheroid and organoid assessments may provide a more accurate understanding of biological switching behaviours as cells transition from tumours to spheroids and back to tumour structures as metastatic lesions. This was highlighted with a transcriptomic analysis that confirmed a dormancy phenotype in spheroids and the importance of cell cycle checkpoint regulation in organoids. Overall, our results provided potentially new mechanistic discoveries that could be targeted therapeutically for EOC.

## 2. Materials and Methods

### 2.1. Cell Lines

The following HGSOC-established cell lines were used for analysis and purchased from ATCC (Cedarlane, Burlington, ON, Canada): OVCAR8, OVCAR4 and OVCAR3 (Table 1). The following HGSOC patient ascites-derived immortalized cell lines were used: iOvCa182, iOvCa195, iOvCa198, iOvCa246, iOvCa256, iOvCa398 and iOvCa411 (Table 1). iOvCa cell lines were derived from the malignant ascites fluid collected during debulking surgery and continuously passaged on T-75 flasks (Sarstedt, Newton, NC, USA) until immortalized [22]. Patient consent for the clinical specimens from which these cell lines were derived was obtained according to our institution’s research ethics board-approved protocol (#115904). The cells were maintained at 37 °C and a CO_2_ concentration of 5% in adherent cultures on tissue culture-treated polystyrene plates (Sarstedt, Newton, NC, USA) with 10% foetal bovine serum (FBS) (Wisent, Saint-Jean-Baptiste, QC, Canada) in either RPMI-1640 (Gibco, Grand Island, NY, USA) for OVCAR8 and OVCAR3 cell lines, or DMEM/F-12 (Gibco) for OVCAR4 and iOvCa cell lines. All cell lines were confirmed to be HGSOC based on their marker expression of PAX8 and p53 (Figure S1A,B; Table 1). All cell lines were validated by STR analysis (The Centre for Applied Genomics, The Hospital for Sick Children, Toronto, ON, Canada) and tested for mycoplasma routinely using the Universal Mycoplasma Detection Kit (ATCC; Cedarlane, Burlington, ON, Canada).

### 2.2. Three-Dimensional Spheroid Culture

The cells were seeded at 5 × 10^5^ or 1 × 10^6^ cells/well in suspension using 6-well ULA plates (Corning, New York, NY, USA) and supplemented with their corresponding media (RPMI-1640 or DMEM/F12 with 10% FBS). The spheroids were collected after 3 days in culture.

### 2.3. Three-Dimensional Organoid Culture

The cells were seeded at 2.5 × 10^5^ or 5 × 10^5^ cells/well and resuspended in 50 µL of Cultrex Basement Membrane Extract (BME) PathClear Type 2 (Cedarlane, Burlington, ON, Canada) as droplets on 24-well tissue culture-treated polystyrene plates (Sarstedt, Newton, NC, USA). The droplets were overlaid with EOC organoid-specific media containing Advanced DMEM/F-12 (Invitrogen, Waltham, MA, USA) and supplemented with B-27™ (Invitrogen, Waltham, MA, USA), Forskolin (Cedarlane, Burlington, ON, Canada), GlutaMAX™ (Invitrogen, Waltham, MA, USA), HEPES (Wisent, Saint-Jean-Baptiste, QC, Canada), Human EGF (Peprotech Inc., Cranbury, NJ, USA), Human FGF-10 (Peprotech Inc., Cranbury, NJ, USA), Nicotinamide (MilliporeSigma, Oakville, ON, Canada), N-Acetyl-L-cysteine (MilliporeSigma, Oakville, ON, Canada), Recombinant Human Noggin (R&D Systems, Toronto, ON, Canada) and Y-27632 dihydrochloride (MilliporeSigma, Oakville, ON, Canada) (Formula provided in Table S1). After 21 days in culture, the organoids were released from Cultrex BME by dissolving the matrix dome in 500 µL of Cell Recovery Solution (Corning, New York, NY, USA) incubated on ice for 1.5–2 h.

### 2.4. Organoid Growth Analysis

The cells were seeded at 5 × 10^4^ cells/well in 50 µL of Cultrex BME on a 24-well tissue culture plate (Corning, New York, NY, USA). For manual analysis, brightfield images were taken at the 21-day endpoint using a Leica DMI4000B inverted microscope (Teaneck, NJ, USA). ImageJ Software v1.54m was used to manually outline each organoid structure to quantify organoid number (count/well or image) and average organoid area (µm^2^). For automated analysis, the Incucyte^®^ S3 System (Sartorius, Oakville, ON, Canada) was used to capture brightfield images at 12 h increments over 21 days. The Incucyte^®^ Organoid Analysis Software Module v2023B (Sartorius, Oakville, ON, Canada) was used to automatically outline each organoid structure to quantify the total organoid area per image or well (µm^2^), organoid number, organoid darkness and organoid eccentricity.

### 2.5. Antibodies

The following primary antibodies, from Cell Signaling Technologies (Whitby, ON, Canada), were utilized at 1:1000 dilution: phosphorylated AMPKα at T172 (2535), AMPKα (5832), phosphorylated Akt at S473 (4060), Akt (9272), AURKA (14475), phosphorylated cdc2 at Y15 (CDK1, 4539), cdc2 (CDK1, 9116), phosphorylated cdc25C at S216 (4901), cdc25C (4688), phosphorylated γH2A.X at S139 (9718), cleaved PARP (9541), p53 (2527), PCNA (2586), PLK1 (4513) and WEE1 (13084). The antibodies for vinculin (V9264, 1:40,000) and actin (A2066, 1:20,000) were purchased from MilliporeSigma (Oakville, ON, Canada). The antibody for PAX8 (10336-1-AP, 1:500) was purchased from Proteintech (Rosemont, IL, USA). The HRP-conjugated secondary antibodies used were anti-rabbit IgG (NA934V, 1:10,000), anti-mouse IgG (NA931V, 1:10,000), or anti-rabbit FITC (F9887, 1:300) from MilliporeSigma (Oakville, ON, Canada).

### 2.6. Protein Isolation

The adherent cells at 80% confluency were washed with PBS and scraped into a modified radioimmunoprecipitation assay (RIPA) lysis buffer solution. Spheroids and organoids were collected into 15 mL or 50 mL tubes, washed with cold PBS, spun down and then lysed using the same lysis buffer. The cells were stored at −80 °C until the next day and then lysed with vortexing every 5 min. The lysates were centrifuged (21,000× *g*, 4 °C, 20 min) and the supernatant was collected for protein analysis.

### 2.7. Immunoblotting

The protein lysates were prepared at 15–30 μg per sample being mixed with modified RIPA buffer and resolved on 8–12% polyacrylamide SDS gels by electrophoresis at 100 volts and 120 volts each for 1 h (a total of 2 h running time) in running buffer. The proteins were transferred to a PDVF membrane (Bio-Rad, Mississauga, ON, Canada) for 1 h at 100 volts in transfer buffer. The membranes were blocked with 5% skim milk or BSA in Tris-buffered saline-Tween 20 (TBST) for 1 h at room temperature. The protein-specific primary antibodies were diluted in 5% BSA + TBST, except for p53 and cdc2, which used 5% skim milk + TBST and incubated overnight at 4°C with gentle agitation. The membranes were incubated for 1 h at room temperature with a secondary antibody in 5% BSA + TBST. Protein detection was achieved through a chemiluminescence reagent, and imaging was performed using the Chemidoc™ MP 7 System (Bio-Rad, Mississauga, ON, Canada). Densitometric analyses were performed using ImageLab™ 6.1 Software (Bio-Rad, Mississauga, ON, Canada).

### 2.8. Hematoxylin and Eosin Staining

Intact spheroids and organoids were washed with cold PBS and then fixed with 10% Formalin for 15 min. The pellets were resuspended in 3% agarose in PBS, polymerized at 4 °C overnight, then placed into cassettes and processed through graded alcohols to paraffin. The embedded pellets in paraffin blocks were cut at 5 µm sections onto charged slides (Thermo Fisher Scientific, Mississauga, ON, Canada), and H&E staining was performed. Images of H&E-stained sections were captured using an Aperio ScanScope slide scanner (Leica, Teaneck, NJ, USA) and exported using the Aperio ScanScope Console v101.0.4.413 (Leica, Teaneck, NJ, USA).

### 2.9. Immunohistochemistry

Previously archived formalin-fixed, paraffin-embedded blocks were sectioned at a thickness of 5 μm. Immunohistochemical staining for Antigen Kiel 67 (Ki67) with hematoxylin counterstaining was completed. Sectioning and staining were performed by the Molecular Pathology Core Facility at the Robarts Research Institute (London, ON, Canada). Images of IHC-stained sections were captured using an Aperio ScanScope slide scanner (Leica, Teaneck, NJ, USA). Images of individual stained sections were exported using the Aperio ScanScope Console v101.0.4.413 (Leica, Teaneck, NJ, USA). Using ImageJ Software, the imaged slides were analyzed for staining intensity. The nuclei were masked using the Trainable Weka Segmentation plugin, and the masked regions were counted using a minimum area of 100 and a maximum area of 1000 pixels^2^. Each pixel was graded based on the staining intensity as high positive (0–60), positive (60–120), low positive (120–180) and negative (>180) groups.

### 2.10. Immunofluorescence

Intact spheroids and organoids were collected into 50 mL conical tubes and allowed to settle passively. Using Cryomatrix (Thermo Fisher Scientific, Mississauga, ON, Canada), the pellets were embedded in a 15 × 15 × 5 mm disposable base mold (Thermo Fisher Scientific, Mississauga, ON, Canada). The blocks were cryosectioned at 7 µm onto charged slides and the tissues were fixed using 4% Neutral Buffered Formalin (Thermo Fisher Scientific, Mississauga, ON, Canada), permeabilized with 0.1% Triton-X100 in PBS and blocked with 5% BSA in 0.1% Triton-X100 PBS. The primary antibodies were diluted in blocking solution and incubated overnight at 4 °C. The secondary antibody was diluted in blocking solution and incubated for 1 h at room temperature. Alexa Fluor Phalloidin (Thermo Fisher Scientific, Mississauga, ON, Canada), at 1:40 dilution, was incubated for 1 h, then slides were mounted using VectaShield (Vector Laboratories, Newark, CA, USA) containing a 1:1000 dilution of DAPI (MilliporeSigma, Oakville, ON, Canada). Fluorescent images were captured using the Olympus AX70 upright microscope and ImagePro Software 10.0.15.

### 2.11. RNA Isolation

The spheroids and organoids were collected into 15 mL or 50 mL tubes, washed with cold PBS and pelleted. RNEasy Spin Column Kit (Qiagen, Toronto, ON, Canada) was used to isolate RNA, and genomic DNA was removed by RNase-free DNaseI (Qiagen, Toronto, ON, Canada), both using the manufacturer’s instructions. RNA concentrations, A_260/280_ and A_260/230_, were measured using the NanoDrop Microvolume Spectrophotometer (Thermo Fisher Scientific, Mississauga, ON, Canada).

### 2.12. Transcriptomic Analysis

For RNA sequencing analysis, isolated RNA from iOvCa spheroid and organoids (n = 7) were collected in duplicate and sent to the London Regional Genomics Centre at the Robarts Research Institute for library preparation. The RNA quality was first confirmed using 2100 Bioanalyzer Instrument (Agilent Technologies, Mississauga, ON, Canada), and sequencing was performed on an Illumina NextSeq using High Output 75 cycle sequencing kits. Raw sequencing reads were uploaded to the Galaxy platform and tested for quality using FastQC and MultiQC to measure the per base sequence quality, GC content, N content, overrepresented sequences and adapter content. The reads were mapped to the human genome (hg38) using HISAT2. The differential gene expression was determined using DESeq2. Pathway analysis was performed by gene set enrichment analysis (GSEA) using all gene set collections from the Molecular Signatures Database, focusing on the Cancer Hallmark and Curated gene sets.

### 2.13. Quantitative Reverse Transcription-(qRT)-PCR

Complementary DNA (cDNA) was generated from total RNA using the HighCapacity cDNA Reverse Transcriptase Kit (Thermo Fisher Scientific, Mississauga, ON, Canada) according to the manufacturer’s instructions. The reactions were completed using 1 μg of RNA while using the MyCycler thermocycler (Bio-Rad, Mississauga, ON, Canada) with the following cycles: 25 °C for 10 min, 37 °C for 120 min, 85 °C for 5 min and 4 °C until sample retrieval. qRT-PCR was performed using the Brilliant II SYBR Green Master Mix (Agilent Technologies, Mississauga, ON, Canada) according to the manufacturer’s instructions. The reactions were performed using a QuantStudio3 RT-PCR system (Thermo Fisher Scientific, Mississauga, ON, Canada), and data analysis was performed using the QuantStudio Design and Analysis Software v1.4.3 with the 2^−ΔΔCT^ method for relative fold-change calculations. The primers were purchased from Invitrogen (Waltham, MA, USA) (Table S1).

### 2.14. Kinase Inhibitor Treatments

Adavosertib (10 mM), Alisertib (20 mM) and Volasertib (10 mM) were purchased from Cedarlane (Burlington, ON, Canada), reconstituted in dimethylsulfoxide (DMSO) and stored at −20 °C until use. The adherent cells were seeded at 2 × 10^4^–6 × 10^4^ cells/well on 96-well standard tissue culture plates and treated 24 h after plating for dose response curves. For spheroids and organoids, the cells were seeded at 5 × 10^4^ cells/well in 96-well round bottom ULA plates and in 10 µL of Cultrex^®^ BME on 96-well standard tissue culture plates with their corresponding media types and left for 3 days. The cells were treated with a 3-fold serial dilution of Adavosertib (0.001–10 µM), Alisertib (0.01–100 µM) or Volasertib (0.001–10 µM) for 72 h. An alamarBlue™ assay (Thermo Fisher Scientific, Mississauga, ON, Canada) was completed for cell viability, and raw data were normalized to DMSO (Adavosertib and Volasertib) or the lowest drug concentration (Alisertib) as the control.

### 2.15. Statistical Analysis

The graphs were generated using GraphPad Prism 10 (GraphPad Software, San Diego, CA, USA), and most data were expressed as mean ± SEM. Student’s *t*-test, an ordinary one-way ANOVA, or a two-way ANOVA with Tukey’s multiple comparisons test was performed, with results being considered significant at *p* < 0.05. Specific details on graphs, statistical tests and significance values are provided in each figure legend.

## 3. Results

### 3.1. HGSOC Cell Lines Showed Distinct Spheroid and Organoid Morphologies

**Figure 1:**
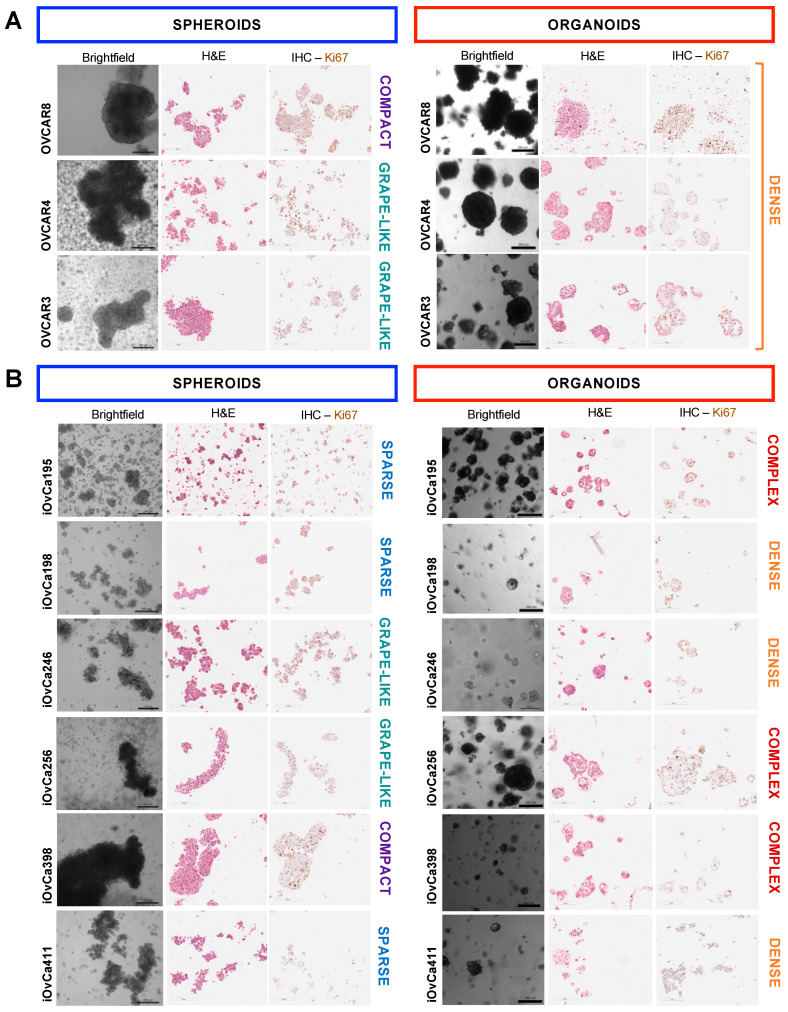
Morphological and histological comparisons of HGSOC cell lines cultured using 3D model systems. (**A**,**B**) Brightfield images of established cells lines and patient ascites-derived immortalized cell lines cultured as spheroids and organoids. The structures were categorized based on appearance as compact, grape-like, or sparse spheroids, and dense or complex organoids, as indicated on the right side of each cell line-associated image. Spheroids and organoids were also collected for formalin-fixed paraffin-embedding (FFPE), sectioning and staining for H&E and IHC with the Ki67 proliferation marker. The scale bar is 200 µm or 250 µm for brightfield, and 200 µm for H&E and IHC images.

To validate our culture systems for model development, three established HGSOC cell lines (i.e., OVCAR3, OVCAR4 and OVCAR8) were grown as spheroids on ULA plates and as organoids in Cultrex© BME with an EOC organoid specific media (Table S1). This was important in developing the appropriate culture conditions for the organoid model system in comparison to our suspension spheroid model. Brightfield imaging, as well as hematoxylin and eosin (H&E) staining on sections was conducted for these OVCAR cells lines for a morphological analysis of individual structures (Figure 1A). We characterized the spheroid morphologies based on the density of cellular aggregation as compact, grape-like or sparse clusters [22,25,39]. As expected, OVCAR8 cells formed large, compact spheroids, whereas OVCAR4 and OVCAR3 cells formed large, grape-like clusters as observed previously when studying HGSOC spheroid formation (Figure 1A). Comparatively, we characterized organoids as dense structures that appeared solid or complex structures that appeared as a disorganized mixture of cystic formations and cell clumps [28,40]. All the OVCAR cell lines formed similarly large, dense organoids. This uniform morphology was unexpected to other studies on EOC PDOs, indicating a lack of heterogeneity within these established cell lines.

Thus, we incorporated our own patient ascites-derived immortalized cell lines (iOvCa) into this analysis. These cell lines were developed from patient ascites samples and passaged on standard tissue culture plates until immortalisation, as previously described [22]. Identical methods were conducted using six iOvCa cell lines after being cultured in these two model systems (Figure 1B). This was important to establish how these particular cells appear, grow and behave within these models, as they have not been widely used in our previous research. Initially, we noted that the iOvCa spheroids and organoids appeared very heterogeneous with varying sizes and morphologies, as seen in the brightfield and H&E-stained images (Figure 1B). Using the same categorisations above, the iOvCa398 cells appeared as compact spheroids, and the iOvCa246 and iOvCa256 cells adopted a grape-like structure. The remaining three cell lines (i.e., iOvCa195, iOvCa198 and iOvCa411) formed sparse clusters with abundant cellular debris, indicating some cell death during spheroid formation [41,42]. Again, the iOvCa organoids varied, where half of the cell lines appeared as dense (i.e., iOvCa198, iOvCa246 and iOvCa411) and the other half appeared complex with a sporadic mix of dense and cystic structures (i.e., iOvCa195, iOvCa256 and iOvCa398). In addition, iOvCa256 and iOvCa398 organoids appeared to have necrotic cores as seen upon H&E staining (Figure 1B). Overall, the spheroid and organoid morphologies largely varied, where the iOvCa cell lines better portrayed HGSOC heterogeneity than the OVCAR cell lines, and the organoids better resembled PDOs, as published. Hence, we focused our analyses on the iOvCa cell lines moving forward.

### 3.2. HGSOC Organoid Varying Growth Dynamics Relate to Morphology

Since our previous 3D models using spheroids in suspension have shown a dormancy phenotype in comparison to 2D adherent culture, we wanted to determine whether spheroids differ to organoids in a similar manner. Also, organoids have become a popular model system in cancer research, and it is imperative to understand organoid growth dynamics in the context of EOC, which is not well known. The results showed that the iOvCa cell lines demonstrated great heterogeneity with organoid growth. The iOvCa195 cells formed the most organoids and the iOvCa198 cells formed the least after 3 weeks of culture (Figure S2A). The largest organoids were greater than 20,000 µm^2^ in individual organoid area (i.e., iOvCa246), the smallest were less than 5000µm^2^ (i.e., iOvCa198, iOvCa398 and iOvCa411), and the rest had an organoid size in between 10,000 and 15,000 µm^2^ in area (i.e., iOvCa195 and iOvCa256) (Figure S2B). Although when observing growth over time, the total organoid number often reached a maximum by day 3 in culture, indicating that the capacity to form an organoid was established by that time point (Figure S2E). As predicted, the total organoid area had a steady increase from day 1 for the iOvCa195, iOvCa246 and iOvCa411 cells, yet the other three cell lines plateaued after days 3–6 in culture (Figure S2F).

**Figure 2:**
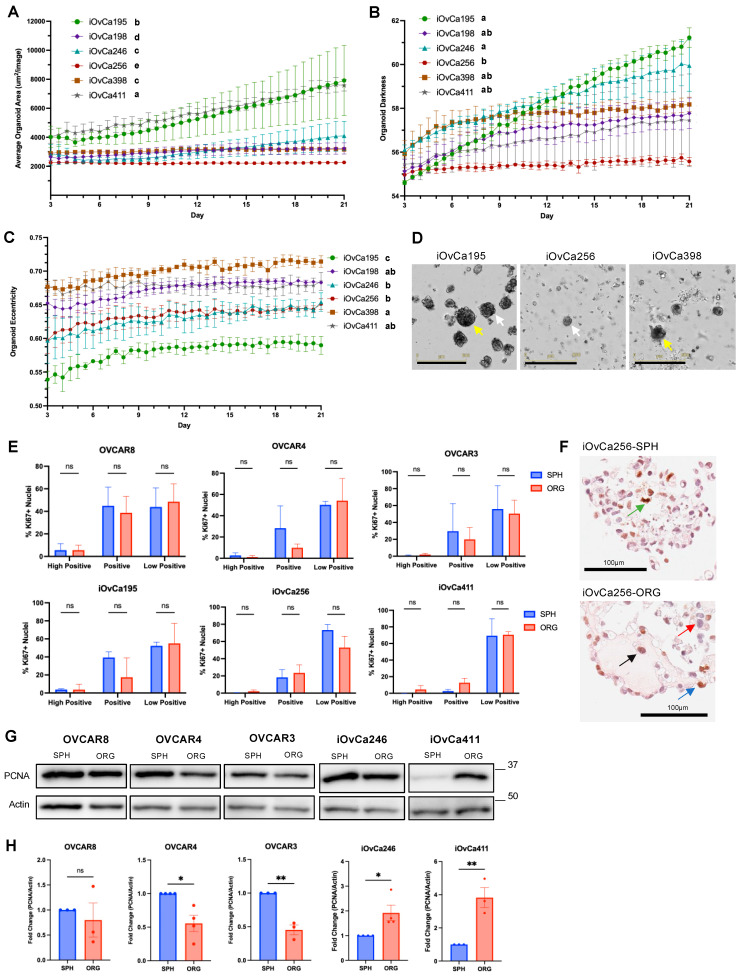
Growth analysis in relation to proliferation status of HGSOC spheroids and organoids. (**A**) Average organoid number (μm^2^/image) over time was calculated using organoid number and total organoid area measurements. (**B**) Organoid darkness was measured to observe the density of cells or debris within a structure over time. (**C**) Organoid eccentricity was measured to observe the difference in shape over time. (**D**) Representative brightfield images of iOvCa195, iOvCa256 and iOvCa398 organoids at day 21, with white arrows for organoid darkness and yellow arrows for organoid eccentricity. The scale bar is 600 µm. (**E**) The IHC sections were used to measure the percentage of Ki67 positive (Ki67+) nuclei, and they were binned based on pixel intensity within ImageJ for each indicated cell line (n = 2–3). (**F**) High magnification images of representative organoids with arrows indicating high positive (green), positive (blue), low positive (black), Ki67+ nuclei and Ki67- nuclei (red). (**G**) Immunoblots of PCNA were completed to confirm the proliferative index of spheroids versus organoids. (**H**) Relative quantification of PCNA in spheroids (SPH) and organoids (ORG). The graphs were generated using GraphPad Prism 10 demonstrating mean ± SD for (**E**) and mean ± SEM for (**A**–**C**,**H**). Statistical analysis for (**A**) was completed on the simple linear regression curve of each cell line over all timepoints; statistical analysis for (**B**,**C**) was completed on only the Day 21 data, both using an ordinary one-way ANOVA with Tukey’s multiple comparisons test and displayed with compact lettering for each cell line, indicated as bold letters; statistical analysis for (**E**,**H**) was a Student’s *t* test for each cell line (n ≥ 3, ns = not significant, * *p* < 0.05, ** *p* < 0.01).

Furthermore, we calculated the average organoid area based on organoid number and total organoid area measurements, which demonstrated that each cell line had its own unique linear growth rate (Figure 2A; Video S1). Of note, the iOvCa195 and iOvCa411 lines had the greatest increase in average organoid area over time. All iOvCa growth rates, based on a simple linear regression analysis, were significantly different from each other, except for iOvCa246 and iOvCa398, which both had a similar morphology. Moreover, we incorporated organoid darkness and organoid eccentricity characteristics as another way to quantify HGSOC organoid morphology. Organoid darkness gradually increased over time in each cell line, with significantly different endpoint values. Correlating these values to morphology, iOvCa246 organoids had the second highest darkness value (59.9 ± 1.2), which could indicate a dense morphology; in contrast, iOvCa256 organoids had the lowest value (55.6 ± 0.2) in conjunction with a complex morphology and necrotic centre (white arrows; Figure 2B,D). However, the other four cell lines had inconsistent darkness values that did not match their designed morphology. The eccentricity of iOvCa organoids also gradually increased over time for each cell line, indicating that the organoids become more irregularly shaped as they grow (Figure 2C). The cell lines were grouped based on their endpoint organoid eccentricity values to categorize them into two shapes: those with a value greater than 0.6 had an irregular shape, like iOvCa398 organoids with the highest value of 0.714 ± 0.008, and those with a value of less than 0.6, like iOvCa195 organoids with the lowest value of 0.590 ± 0.013 (yellow arrows; Figure 2C,D), were regular. Taken together, there is clear heterogeneity in iOvCa organoid growth characteristics that can easily be quantified through various methods. In contrast, the OVCAR cell lines had the largest organoids based on number and size after 3 weeks of growth (Figure S2A–C). With growth over time, the OVCAR cell lines had no significant differences with organoid number, total organoid area and average organoid area (Figure S3A–C). However, OVCAR4 did have a higher darkness value, which is apparent in the brightfield images (Figure S3D,F). Based on the organoid growth characteristics, this indicates that the OVCAR cells lines are not an accurate representative model for observing the heterogeneity seen in HGSOC.

To gain more knowledge on the 3D structure of spheroids and organoids, quantification of Ki67 immunohistochemistry (IHC) was completed to assess cell proliferation. Ki67 staining was grouped based on pixel intensity as high positive (0–60), positive (60–120) and low positive (120–180). Unexpectedly, there were no significant differences in Ki67-positive (Ki67+) nuclei between spheroids and organoids in the different Ki67 staining intensity groups (Figure 2E,F). To further validate these findings, immunoblots were conducted for proliferative cell nuclear antigen (PCNA). The results showed that iOvCa246 and iOvCa411 organoids had higher PCNA protein levels than their spheroids (Figure 2G,H). However, the remaining iOvCa cell lines showed no significant differences in PCNA levels, matching the Ki67 staining data (Figure S2G,H). Interestingly, the OVCAR4 and OVCAR3 spheroids had higher PCNA protein levels than their organoids (Figure 2G,H), reaffirming their deviation from patient-associated HGSOC phenotypes. Overall, these results, using two standard cell proliferation markers, indicate that there are very little differences between spheroids and organoids in their proliferation status.

### 3.3. Altered AMPK and Akt Signaling in HGSOC Spheroids and Organoids Indicates Biological Switching

**Figure 3:**
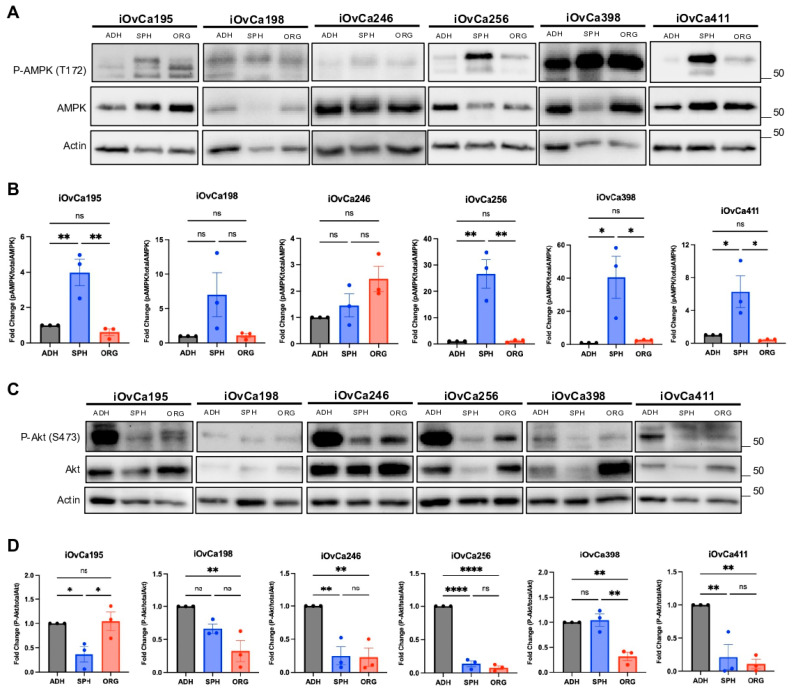
Altered phosphorylation status of AMPK and Akt between culture model systems. (**A**,**B**) Relative protein expression and quantified fold change in phosphorylated AMPK at T172 (P-AMPK) to total AMPK in adherent (ADH), spheroid (SPH) and organoid (ORG) cultures. (**C**,**D**) Relative protein expression and quantified fold change in phosphorylated Akt at S473 (P-Akt) to total Akt in adherent (ADH), spheroid (SPH) and organoid (ORG) cultures. The graphs were generated using GraphPad Prism 10 demonstrating mean ± SEM and a one-way ANOVA with Tukey’s multiple comparisons test for statistical analysis of each individual cell line (n = 3, ns = not significant * *p* < 0.05, ** *p* < 0.01, **** *p* < 0.0001).

Previously, we determined the pathways involved in spheroid formation, including cellular quiescence, bioenergetic stress and autophagy, which indicated a switch to dormancy [13,14]. However, we only have knowledge on the molecular differences between adherent and spheroid models and have not assessed these pathways in organoids. As of now, it is not clear whether organoids may differ from spheroids in terms of proliferation and growth. Thus, we observed AMPK, a known kinase that is highly active during the bioenergetic stress response and is required for cell survival during spheroid formation [18,22]. The iOvCa cell lines showed >4-fold increase in phosphorylated-AMPK (P-AMPK) levels at T172 in spheroids compared to adherent and organoid cultures (Figure 3A,B). We noted that the total AMPK protein levels fluctuated between spheroid and organoid samples, which could exacerbate this effect in some samples. The net result, however, supports the notion of an increased requirement for active AMPK within spheroids for cell survival, as we demonstrated previously [18,22]. The exceptions were the iOvCa198 and iOvCa246 cells, with no significant difference in levels of P-AMPK in all culture conditions, but the iOvCa198 cells seemed to have followed a similar trend with potentially higher levels in spheroids. To address whether differences in culture media composition may contribute to signaling changes, particularly with the additional supplements in the EOC organoid specific media, we cultured spheroids in this medium to assess AMPK protein levels. Indeed, we observed similar P-AMPK protein levels between spheroids grown in each medium, indicating that at least this difference in culture conditions has no effect on AMPK pathway activity (Figure S4). This suggests that AMPK is important during spheroid formation in the majority of iOvCa cell lines, and not necessarily for organoid growth. Conversely, we saw a dramatic increase (>10-fold) in P-AMPK levels in the OVCAR8 and OVCAR4 organoids compared to adherent cultures, while still having high levels in spheroids (Figure S3G,H). Also, the OVCAR3 cells had no significant differences between any culture models, similar to iOvCa198 cells. This reiterates that the iOvCa cell lines better demonstrate HGSOC molecular signaling changes.

Next, we interrogated the phosphorylation status of Akt, a prominent kinase downregulated during spheroid formation, as cells switch from a proliferative phenotype to a quiescent or dormant phenotype [25,43]. As predicted, there was a significant decrease in phosphorylated-Akt (P-Akt) at S473 in most iOvCa cell lines from adherent to spheroid cultures, except in the iOvCa198 and iOvCa398 lines, which had similar levels (Figure 3C,D). Interestingly, the iOvCa195 cells showed no significant difference in P-Akt in organoids compared to adherent cultures, while the rest had decreased levels in organoid cultures. Furthermore, only the iOvCa195 and iOvCa398 cell lines showed significant differences between their spheroid and organoid P-Akt levels, but with opposites trends (Figure 3C,D). Similarly, the OVCAR cell lines all showed decreased P-Akt levels from adherent to spheroid cultures, but varying levels in organoids (Figure S3I,J). The OVCAR8 and OVCAR3 levels showed similar spheroid and organoids levels, while the OVCAR4 organoids had higher P-Akt levels compared to both levels in adherent and spheroid cells. In conclusion, these results display the different molecular signaling activity between our HGSOC spheroids and organoids and demonstrate the potential of HGSOC cells to transition their cell-signaling behaviours depending on their 3D culture state. Based on the above results, there is a lack of consistency in the differences between spheroid and organoid molecular signaling activity in terms of dormant to proliferative switching. Therefore, a comprehensive transcriptomic analysis may better elucidate the underlying biology of HGSOC spheroids and organoids.

### 3.4. Differential Gene Expression Between HGSOC Spheroids and Organoids

**Figure 4:**
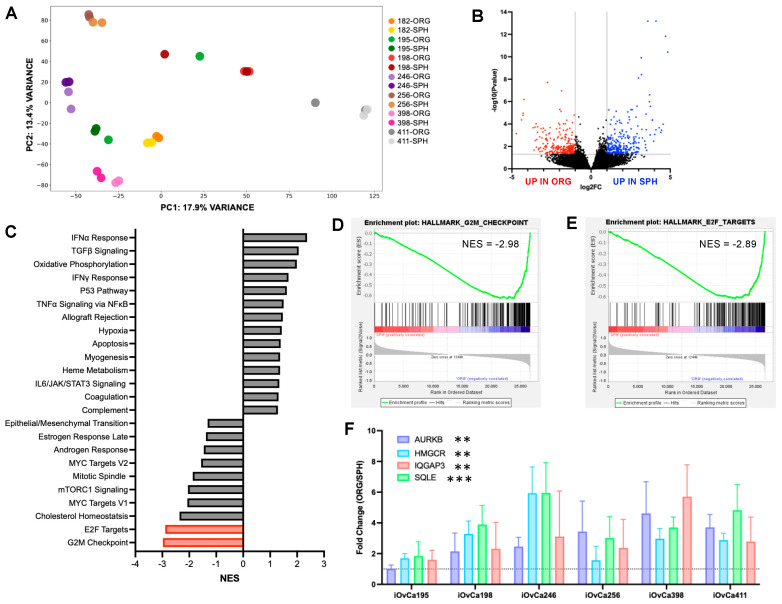
Discovery-based analysis of HGSOC spheroids and organoids based on bulk RNA-sequencing results. (**A**) Principal component analysis plot of all samples, where each colour indicates a cell line and each hue change differentiates spheroids (SPH) from organoids (ORG). (**B**) Differential gene expression between spheroids and organoids with the coloured dots (blue—elevated in organoids, red—elevated in spheroids) indicating significant genes. (**C**) Normalized enrichment scores (NES) of cancer hallmark gene sets in spheroids (NES = positive) and organoids (NES = negative) with the red bars showing two pathways of interest, G2/M checkpoint and E2F Targets (FDR < 0.05). (**D,E**) Enrichment plots for G2M checkpoint and E2F target grouped gene sets within the cancer hallmark pathways. (**F**) RT-qPCR validation of selected genes within the G2M checkpoint and E2F target cancer hallmark and curated sets, where any bar above the dotted line (fold change = 1) shows an increase. The graphs were generated using GraphPad Prism 10, demonstrating mean ± SEM with a Student’s *t* test for statistical analysis grouping all cell lines to show the difference between spheroids and organoids (n = 3, ** *p* < 0.01, *** *p* < 0.001).

We postulate that there are likely many different intracellular signaling programs altered in spheroids and organoids that could control their growth and morphology beyond those interrogated previously. Hence, we conducted bulk RNA-sequencing analysis on spheroids and organoids using seven iOvCa cell lines. Principal component analysis (PCA) revealed clustering based on cell line rather than culture condition, with only a few single sample outliers seen in iOvCa195-ORG, iOvCa198-SPH and iOvCa411-ORG samples (Figure 4A). This most likely reflects that each cell line recapitulates the original patient source and associated genotype. Differential gene expression analysis was also conducted to identify the top upregulated genes in spheroids and in organoids across all cell lines (Figure 4B). There were a similar number of genes significantly expressed in each model, where 265 were upregulated in organoids, in comparison to 280 upregulated in spheroids. Based on this differential expression, gene set enrichment analysis (GSEA) was completed to identify significantly elevated pathways in the Cancer Hallmarks Collection (Figure 4C). We found fourteen pathways up in spheroids and ten pathways up in organoids (FDR < 0.05). Based on our previous work on HGSOC spheroid formation in comparison to adherent cultures, we anticipated seeing similarly regulated pathways in our iOvCa spheroids. This was confirmed with TGFβ signaling and oxidative phosphorylation Cancer Hallmark pathways being in the top 3 elevated pathways in spheroids [44,45,46].

Since we have copious knowledge on the intercellular signaling changes that occur in spheroids, we pursued the pathways identified in organoids, as our interrogations into pathway analysis is more limited. More specifically, we chose G2/M checkpoint and E2F target pathways because their normalized enrichment scores were the highest, at −2.98 and −2.89, respectively (Figure 4D,E). This was intriguing, as these cancer hallmark pathways are both involved in cell cycle regulation, which we speculate is crucial for actively growing organoids, even though we noted little difference in cell proliferation in previous data [47,48,49]. To further characterize these pathways, validation of the RNA-sequencing results was conducted with genes relevant to the G2/M checkpoint pathway (i.e., *AURKB*, *HMGCR*, and *SQLE*) and E2F target pathway (i.e., *AURKB* and *IQGAP*), either from the Cancer Hallmark or Curated gene sets associated with those pathways. Based on the transcript expression, there is a clear, significant increase in gene expression from spheroids to organoids for nearly all cell lines (log2FC < −1), indicating that these pathways may be important for cell growth in organoids (Figure 4F).

### 3.5. G2/M Checkpoint Is a Key Regulatory Pathway in HGSOC Organoids

**Figure 5:**
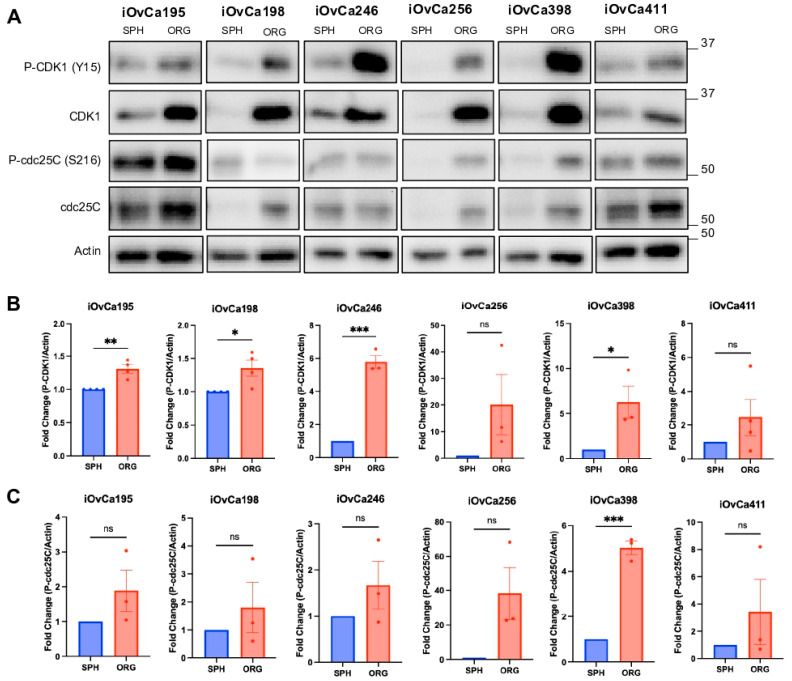
Expression and regulation of G2M checkpoint proteins in HGSOC organoids versus spheroids. (**A**) Relative protein expression of phosphorylated CDK1 at Y15 (P-CDK1), total CDK1, phosphorylated cdc25C at S216 (P-cdc25C) and total cdc25C in spheroid (SPH) and organoid (ORG) cultures. (**B**,**C**) Quantified fold change in P-CDK1 and P-cdc25C to actin housekeeping protein. The graphs were generated using GraphPad Prism 10 demonstrating mean ± SEM a Student’s *t* test for statistical analysis (n ≥ 3, ns = not significant, * *p* < 0.05, ** *p* < 0.01, *** *p* < 0.001).

To confirm that the G2/M checkpoint pathway is important for organoid growth rather than spheroid cell survival, we first assessed the activity of key proteins involved in this pathway. At the G2/M checkpoint, the cell division cycle 25C (cdc25C) was phosphorylated at S216 to sequester it, preventing transition into mitosis [50,51]. When the cells are ready to progress into mitosis, active cdc25C dephosphorylates cyclin-dependent kinase 1 (CDK1) at Y15 so that it can bind to Cyclin B1 for the activation of mitosis. Thus, we determined the baseline levels of phosphorylated-CDK1 (P-CDK1) and phosphorylated-cdc25C (P-cdc25C) in the iOvCa cell lines to indicate proper G2/M checkpoint regulation. The results demonstrated significantly higher levels (>1.25-fold) of P-CDK1 in organoids compared to spheroids, except in the iOvCa256 and iOvCa411 lines (Figure 5A,B). In contrast, only one cell line (i.e., iOvCa398) had increased P-cdc25C levels in organoids compared with spheroids, and the remaining cell lines had a similar trend but without a significant difference (Figure 5A,C). Furthermore, we conducted a preliminary assessment of the expression levels of proteins involved in directly regulating the activity of cdc25C and CDK1, including aurora kinase A (AURKA), polo-like kinase 1 (PLK1) and Mitosis inhibitor protein kinase Wee1 (Wee1). The iOvCa cell lines appear to have differing levels of AURKA and PLK1, but not Wee1, in organoids compared to spheroids (Figure S5). Higher expression of these proteins in organoids suggests elevated G2/M checkpoint cell cycle regulation indicating that functionally targeting these proteins may prove useful.

**Figure 6:**
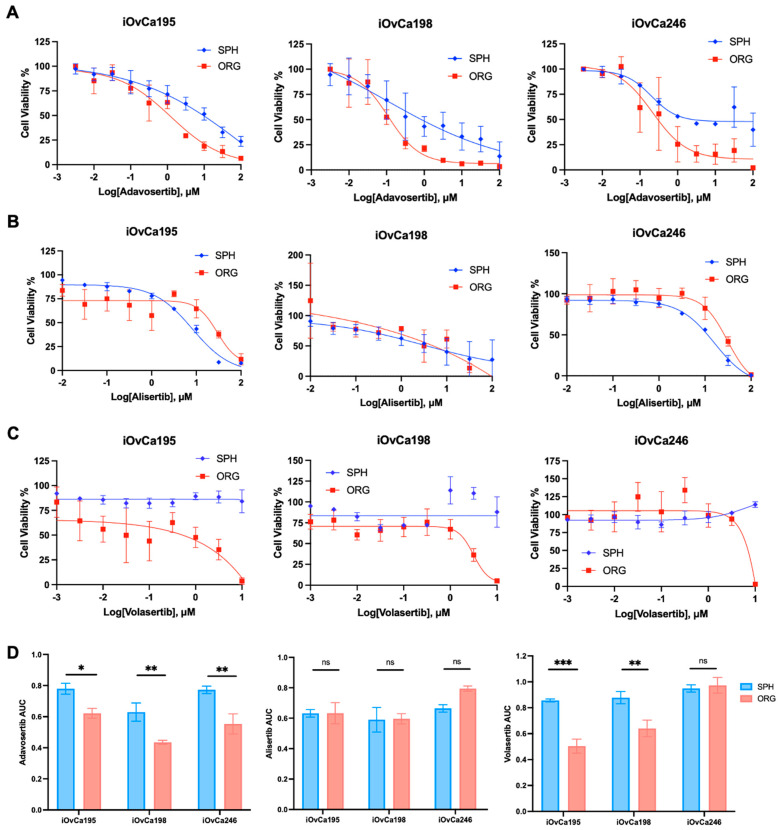
Varying sensitivities to G2M checkpoint small molecule inhibitors on HGSOC spheroid and organoid viability. (**A**–**C**) Dose response curves of a Adavosertib, Alisertib and Volasertib normalized to either DMSO control (Adavosertib and Volasertib) or the lowest drug concentration (Alisertib) based on the alamarBlue readings in spheroid (SPH) and organoid (ORG) cultures. (**D**) Bar graphs of area under the curve (AUC) for each of the above dose–response curves to demonstrate sensitivity between spheroids and organoids. The graphs were generated using GraphPad Prism 10, demonstrating mean ± SEM with two-way ANOVA for statistical analysis (n = 3–4, ns = not significant, * *p* < 0.05, ** *p* < 0.01, *** *p* < 0.001).

To determine if the cells within organoids rely on G2/M checkpoint for cell survival more than within spheroids, we chose to selectively inhibit three regulatory proteins in this pathway (i.e., AURKA, PLK1, Wee1). Blocking CDK1 activity was accomplished with the Wee1 inhibitor, Adavosertib, while impeding cdc25C activity was accomplished with either an AURKA inhibitor, Alisertib, or a PLK1 inhibitor, Volasertib [52,53,54]. All these inhibitors have already shown anti-tumour effects in preclinical experiments and have ongoing Phase I/II clinical trials with ovarian cancer patients, either alone or in combination with chemotherapy [55,56]. We first assessed the IC_50_ values for each of the compounds in our lines in standard adherent culture to evaluate our results as compared with previous publications (Figure S6A). The activity of these inhibitors was validated through the accumulation of P-CDK1 and P-cdc25C with treatment of each inhibitor, compared to the DMSO control (Figure S6B). We also observed an increase in the DNA damage marker gamma H2A histone family member X (γH2A.X) and an increase in the apoptosis marker cleaved PARP under treated conditions with the lowest adherent IC_50_ value (Figure S6B). When comparing the spheroid and organoid dose response curves, we found that there are significantly different sensitivities to these inhibitors between model systems. Spheroids were consistently less sensitive to the Wee1 inhibitor Adavosertib than organoids in all three iOvCa cell lines that we tested (Figure 6A). Additionally, there were no cytotoxic effects on spheroids treated with the PLK1 inhibitor Volasertib as compared to organoids, except for iOvCa246 cells, which showed no difference in sensitivity between spheroids and organoids (Figure 6C). However, when spheroids and organoids were treated with the AURKA inhibitor Alisertib, they either had no difference in sensitivity or showed the opposite trend, with organoids being less sensitive than spheroids as observed with the iOvCa198 line (Figure 6B). To quantify and perform statistical analysis of drug sensitivities between spheroids and organoids, we used the area under the curve (AUC) calculations. The AUC for Adavosertib and Volasertib shows a clear significant difference in sensitivity between organoids and spheroids, where spheroids are less sensitive (Figure 6D). Again, the AUC for Alisertib showed no significant difference in sensitivity for the iOvCa195 and iOvCa246 cells, but the opposite trend for the iOvCa198 cells, where spheroids are more sensitive than organoids (Figure 6D). To conclude, G2/M checkpoint regulation is critical for organoid cell survival rather than spheroid cell survival in EOC. This corresponds to our previous results, which indicate that spheroids are more dormant and our organoids are more proliferative. Targeting this biological switch could pose as a novel therapeutic strategy for HGSOC.

## 4. Discussion

HGSOC is known to be a very heterogeneous disease that complicates treatment options for patients [57,58,59]. The unique biological changes that occur during the metastatic process has have been well elucidated in the context of 3D cell–cell interactions. To understand this complex biology, 3D in vitro culture model systems should be incorporated into our research protocols. Here, we employed an EOC organoid model system in conjunction with the spheroid model system. We found that HGSOC heterogeneity is very apparent with our patient ascites-derived immortalized (iOvCa) cell lines. This was expected, as other studies have demonstrated interpatient heterogeneity through single-cell transcriptomics, where HGSOC patient ascites samples cluster by themselves [5,32]. However, there are key proteins and associated signaling pathways that are crucial for cell survival within each model system. This was demonstrated with the bioenergetic stress pathway in terms of increased P-AMPK in spheroids compared to organoids. In addition, our bulk RNA-sequencing analysis provided an extensive list of differentially regulated pathways between spheroids and organoids. One iOvCa cell line (i.e., iOvCa182) was included in our RNA sequencing data but not in our further analysis due to its difficult nature of being maintained in adherent cultures. With our downstream analysis into the G2/M checkpoint pathway, we saw increased sensitivity to G2/M checkpoint-selective inhibitors in organoids compared to spheroids, except with our AURKA inhibitor Alisertib, which could pose as a new therapeutic target for HGSOC. Therefore, these biological differences should be considered when discovering new targeted treatment options for patients, as they may respond differently, depending on their disease progression.

In this study, we employed multiple ways to quantify organoid growth dynamics and correlate them with morphology. Organoid eccentricity is defined as the ability to bud or the irregularity in 3D shape, whereas darkness is defined as the change in organoid brightness over time, which could be associated with an accumulation of cells or debris within 3D structures. The H&E-stained sections best elucidate these organoid characteristics by visualizing their core. The OVCAR cell lines clearly grow more aggressive organoids with increased central cells (i.e., darkness) and active budding for irregularly shaped structures (i.e., eccentricity), whereas the iOvCa cell lines grow more slowly and into round shapes, sometimes with hollow centres, as cystic-like structures. These discrepancies between OVCAR and iOvCa cell lines demonstrates that patient-derived samples better represent the morphology of HGSOC patient tumours, as seen in many other PDO studies [27,28,30]. When comparing organoid to spheroid morphology, the results showed that all iOvCa cell lines had a unique ability to form spheroids and organoids. Moreover, the iOvCa cell lines demonstrated an aggressive morphology, as spheroids do not correlate with an aggressive organoid morphology and vice versa.

Additionally, these 3D morphologies complement the differing AMPK and Akt protein expressions and, more importantly, their activity levels within spheroids and organoids. For example, the iOvCa398 cells formed very small, complex organoids but were able to survive well as spheroids forming large, compact structures. The iOvCa398 compact spheroids correlated with an increase in P-AMPK levels, which we have previously seen, as cells transition into a dormant state for metastasis [18,22,23]. In contrast, the iOvCa195 cells formed medium-sized, complex organoids but formed very small sparse spheroids. We suspected that organoid growth would correspond to high P-Akt levels and lower levels in spheroids, which is displayed with the iOvCa195 line. We believe that these influences in signaling activity may be critical for the growth of organoids and dormancy in spheroids for this cell line. However, it seems that AMPK signaling may be more influential on spheroid formation rather than Akt signaling on organoid growth. Of course, this would need to be investigated further by observing the downstream AMPK and Akt pathway proteins, such as ULK1 for macro-autophagy regulation [17,18,23,25,39,60].

With the development of the organoid model system, we have vastly expanded our ability to study the important cell biology of this disease in a 3D context. Organoids have become a popular research avenue in all cancer fields, as it provides a reproducible way to observe mini tumour-like structures ex vivo [40,61]. For researchers, it is necessary to gain more insight into the 3D interactions within tumours or metastatic lesions to better eradicate HGSOC in patients. Using solely 2D models limits our understanding of cancer cells and their molecular adaptations to survive during disease progression, especially with HGSOC, where it spreads vastly within the peritoneal cavity, as spheroids easily form secondary tumours [62]. This was clearly demonstrated with our immunoblots for P-AMPK and P-Akt, where organoid protein activity levels did not always correlate to adherent culture as speculated. Therefore, this study provides evidence that both the 3D spheroid and organoid model systems should be utilized for EOC research moving forward, as 2D adherent cultures are not accurate to the cellular behaviour in patients.

In conjunction with previously published results, this study confirmed that there are significant changes in protein activity important for the cancer dormancy pathways in HGSOC. Specifically, we provided some insight into the integral signaling pathways for cancer dormancy and biological switching behaviour in EOC. The Ki67 and PCNA quantifications revealed that the spheroids and organoids did not differ in their proliferative capacity in most cell lines. This was an unexpected result but could be due to the fact that Ki67 is expressed in all cell cycles except for G0, indicating an overabundance of Ki67 in all cells within spheroid structures prior to initiating quiescence [63]. Likewise, PCNA functions during DNA replication and repair, making it critical only for the S phase, indicating more than proliferation induction in cells [64]. On the other hand, our transcriptomic analysis demonstrated upregulation in G2/M checkpoint regulation, E2F Targets and MYC targets in organoids compared to spheroids. These results confirmed that there is a difference in proliferative capacity between culture model systems, but with regard to the regulation of cell cycle checkpoints. This indicates that Ki67 and PCNA may not accurately represent the proliferation index of these 3D structures. Since the process cancer dormancy is a complex relationship between decreased proliferation, increased quiescence and resistance to apoptosis, utilizing the appropriate markers is important to understand this interplay during EOC metastasis.

In recent years, researchers have expanded their search for new HGSOC-targeted therapies to include G2/M checkpoint inhibitors. By blocking the G2/M checkpoint, it forces cancer cells into premature mitosis without proper DNA repair; thus, cells accumulate DNA damage, and it triggers cell death via apoptosis [65,66,67]. Given our RNA sequencing data comparing spheroids and organoids, we speculated that each model system would have a different sensitivity to G2/M inhibition. Here, we were able to provide evidence that targeting the G2/M checkpoint pathway is effective. Adavosertib is a well-known Wee1 inhibitor that has been used to inhibit G2/M transition in many other cancers as well as HGSOC [52,68]. Volasertib is a more recently developed selective PLK1 inhibitor that had antitumour activity in platinum-resistant ovarian cancer [69]. Lastly, Alisertib is an AURKA inhibitor that is being tested on solid tumours in Phase I or II clinical trials that result in mitotic catastrophe [53,70,71,72]. Adavosertib and Volasertib clearly showed the difference in sensitivity between model systems where spheroids were less sensitive than organoids regardless of each unique cell line morphology. In contrast, Alisertib showed no difference in sensitivity between spheroids and organoids. This could be due to a shared dependency on AURKA activity within spheroids and organoids independent of cell cycle regulation and mitosis. These other roles include enhancing cancer cell stemness, initiating EMT, or inducing glucose metabolism [73,74,75].

Based on these results, we believe that targeting AURKA could be a new alternative therapy for HGSOC, especially since AURKA is often amplified in ovarian cancer [76]. Alisertib was able to effectively kill cells within dormant spheroids as well as cells within proliferative organoids. We speculate that this may be due to other downstream effects of targeting AURKA in cells or the potential off-target effects of Alisertib. For example, AURKA also regulates centrosome maturation, chromosomal alignment and separation, and assists with the DNA damage response [70,77,78]. Even though Alisertib is highly selective for AURKA, it may affect the activity of Aurora Kinase B, albeit very minimally. Since HGSOC cells often have a large accumulation of genomic instability, inhibiting these processes could lead to mitotic catastrophe, regardless of the biological state. Of course, the full mechanism of action has not been elucidated but could be extrapolated in the future. As an extension of this, we could improve the efficacy of Alisertib in HGSOC by combining it with chemotherapeutics or other targeted agents to overcome chemoresistance or the recurrence of disease.

The few limitations of this study should be considered when interpreting the results. First, the experiments were conducted using cell lines in in vitro systems, which are valuable for controlled mechanistic studies but do not fully recapitulate the complexity of in vivo biological environments. Even though we used recently developed ascites derived patient samples, they were immortalized, which can limit the translation of these findings to physiological conditions [79]. Second, the use of spheroids and organoids introduces additional complexity due to intercellular spatial differences. These three-dimensional models, though more representative of tissue architecture than traditional monolayer cultures, exhibit gradients in oxygen concentration, nutrient availability, and cellular composition across their structure [62,80,81]. We could invoke our new technology of spatial analysis of spheroids (i.e., SPoRTS) and apply it to organoids for a better understanding of these gradients throughout these 3D structures. Therefore, further studies using in vivo models or clinical samples, such as PDOs, are necessary to validate these findings and better understand their relevance in physiological and pathological contexts.

## 5. Conclusions

In conclusion, utilizing in vitro 3D model systems can be an effective way to better understand the biological complexities of EOC. These cumulative results directly correlate with our previous work that describes the biological changes during metastasis in the context of cancer dormancy. It is clear that there are intercellular signaling changes in cells, rather than strictly morphological, which indicates a dormant phenotype in spheroids compared to a proliferative phenotype in organoids. To determine new targeted therapies and their translational applicability to patients, we provide more evidence by using 3D models with patient-derived samples in current research studies.

## Figures and Tables

**Table 1:** Overview of characteristics for HGSOC-established and patient ascites-derived immortalized cell lines.

Cell Line	Histotype	FIGO Staging	CNV Status	Mutations ^†^	p53 Immunoblot Signal	Chemotherapy Prior to Derivation
OVCAR8	HGSC			TP53 (SA) ^#^ KRAS P121H ERBB2 G776V CTNNB1 E26R	Low	N/A
OVCAR4	HGSC			TP53 L130V ^#^	Low	N/A
OVCAR3	HGSC			TP53 R248Q ^#^ PIK3R1 c.1746-2A > T (SA)	High	N/A
iOvCa182	HGSC	IIIB	84.74%	TP53 K132R	High	6 cycles of carboplatin and paclitaxel; trebananib, angiopoietin inhibitor + weekly paclitaxel for 6 months
iOvCa195	Mixed HGSC and Endometrioid	IV	77.89%	TP53 E171fs*61 BRCA1 Y978*	NS	NONE
iOvCa198	HGSC	IIIB	80.37%	TP53 Q192* PIK3CA I391M KDR Q472H	NS	6 cycles of carboplatin and paclitaxel
iOvCa246	HGSC	IIIC	81.09%	TP53 c.560-2A > T (SA)	NS	NONE
iOvCa256	HGSC Endometrial	IIIA	55.63%	TP53 Y220C KIT M541L	Low	4 cycles of carboplatin and weekly paclitaxel
iOvCa398	HGSC	IC	70.88%	n.d.	High	NONE
iOvCa411	HGSC	IIIC	67.84%	TP53 Y234H	High	6 cycles of carboplatin and paclitaxel; 5 cycles doxorubicin; weekly taxol for 8 months; topotecan; etoposide

HGSC—high-grade serous carcinoma. The staging was based on The International Federation of Gynecology and Obstetrics (FIGO) staging system. ^#^ Leroy et al. 2014. Human Mutation [38]. ^†^ Cellosaurus for mutations in OVCAR cell lines; mutations in iOvCa cell lines were determined based on Oncogene Hotspot analysis. SA—Splice Acceptor Mutation; n.d.—Not determined; NS—no signal (refer to Supplementary Figure S1B).

## Data Availability

The data discussed in this publication have been deposited in NCBI’s Gene Expression Omnibus (Edgar et al., 2002) and are accessible through GEO Series accession number GSE284507 [82].

## Supplementary Materials

The following supporting information can be downloaded at: https://www.mdpi.com/article/10.3390/cells14020133/s1, Figure S1: Confirmation of HGSOC histotype in cultured cell lines; Figure S2: Additional organoid growth measurements with proliferation analysis; Figure S3: Spheroid to organoid comparisons in established OVCAR cell lines; Figure S4: The AMPK protein activity is not affected by the supplements added in EOC organoid specific media; Figure S5: Key proteins in the regulation of the G2/M checkpoint pathway; Figure S6: G2/M checkpoint inhibitor activity levels in adherent cultures; Table S1: EOC organoid specific media formula; Table S2: Primer sets for RT-qPCR validation of RNA-sequencing results; Table S3: Summary of results; Video S1: iOvCa195 organoid growth over time at 12 h increments.
