# Supplementary material for: Exploiting Cancer Dormancy Signaling Mechanisms in Epithelial Ovarian Cancer Through Spheroid and Organoid Analysis
Source: Cells. 2025 Jan 17;14(2):133. doi: 10.3390/cells14020133 (PMC11764263; doi:10.3390/cells14020133)
Supplement: Supplementary file 1 [file cells-14-00133-s001.zip › Supplementary Figures and Tables.pdf]

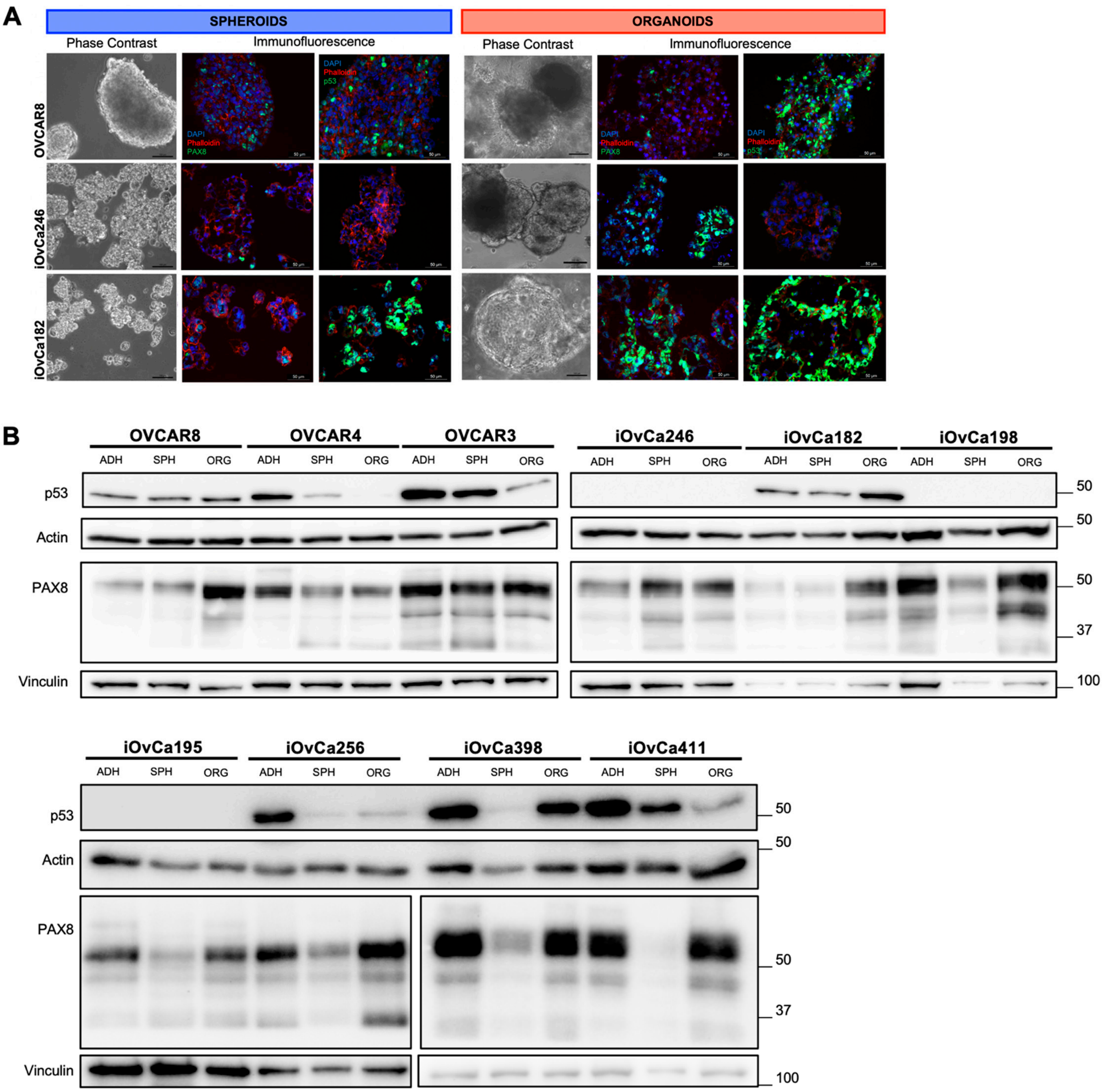

Supplementary Figure S1. Confirmation of HGSOC histotype in cultured cell lines. (**A**) Spheroids and organoids were imaged using phase contrast microscopy at Day 3 and Day 21 end points, respectively. They were collected for OCT cryopreservation, sectioned and immunofluorescent stained for PAX8 and p53. Scale bar is 200μm for phase contrast images and 50μm for IF images. (**B**) Relative protein expression levels of PAX8 and p53 in adherent (ADH), spheroid (SPH) and organoid (ORG) cultures.

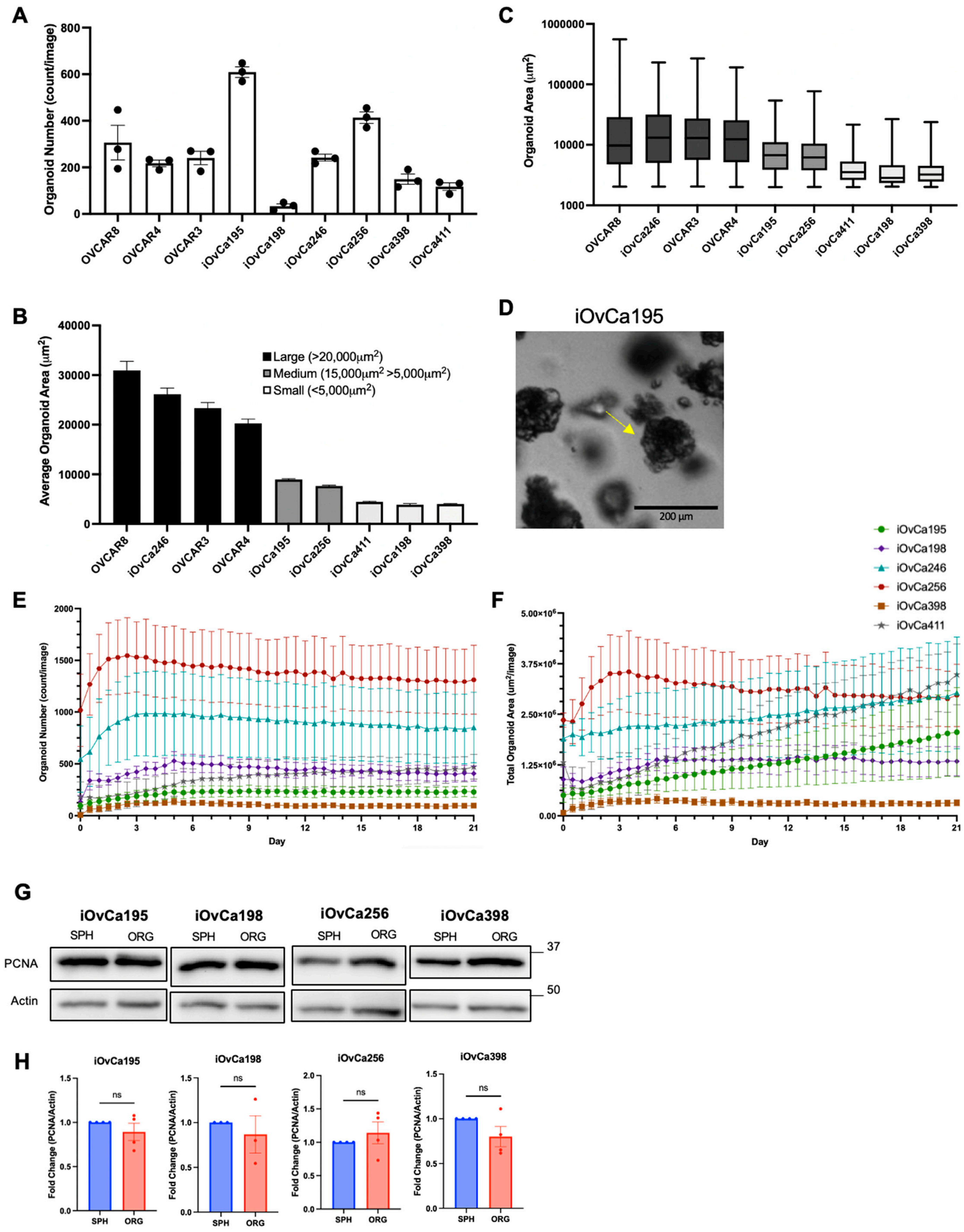
A
Organoid Number (count/image)
800
600
400
200
0
OVCAR8
OVCAR4
OVCAR3
iOvCa195
iOvCa198
iOvCa246
iOvCa256
iOvCa398
iOvCa411
C
Organoid Area (µm²)
1000000
100000
10000
1000
OVCAR8
iOvCa246
OVCAR3
OVCAR4
iOvCa195
iOvCa256
iOvCa411
iOvCa198
iOvCa398
B
Average Organoid Area (µm²)
40000
30000
20000
10000
0
Large (>20,000µm²)
Medium (15,000µm² >5,000µm²)
Small (<5,000µm²)
OVCAR8
iOvCa246
OVCAR3
OVCAR4
iOvCa195
iOvCa256
iOvCa411
iOvCa198
iOvCa398
D
iOvCa195
200 µm
iOvCa195
iOvCa198
iOvCa246
iOvCa256
iOvCa398
iOvCa411
E
Organoid Number (count/image)
2000
1500
1000
500
0
0
3
6
9
12
15
18
21
Day
F
Total Organoid Area (um²/image)
5.00×10⁶
3.75×10⁶
2.50×10⁶
1.25×10⁶
0.00
0
3
6
9
12
15
18
21
Day
G
iOvCa195
iOvCa198
iOvCa256
iOvCa398
SPH
ORG
SPH
ORG
SPH
ORG
SPH
ORG
PCNA
Actin
37
50
H
iOvCa195
iOvCa198
iOvCa256
iOvCa398
Fold Change (PCNA/Actin)
1.5
1.0
0.5
0.0
2.0
ns
SPH
ORG

Supplementary Figure S2. Additional organoid growth measurements with proliferation analysis. (**A,B,C**) Each organoid was manually outlined in brightfield images using the ImageJ Software to provide data on the organoid number, the average organoid area, and the range of varying organoid areas within a cell line at the 21-day growth endpoint. (**D**) Brightfield image of iOvCa195 organoids with yellow arrow representing an average sized organoid for this cell line. Scale bar is 200μm. (**E,F**) Incucyte® S3 software automatically identified each organoid and outlined its border to measure organoid number and total organoid area per image or well. (**G)** Immunoblots of PCNA were completed to confirm the proliferative index of spheroids versus organoids. (**H**) Relative quantification of PCNA in spheroids (SPH) and organoids (ORG). Graphs were generated using GraphPad Prism 10. Graphs for A and B are shown in mean±SD. Graph for C with box showing 25th percentile to 75 percentile and median as line in the middle, and whiskers showing minimum and maximum value. Graphs for E, F and H show mean±SEM with a Student's t test for statistical analysis (n≥3).

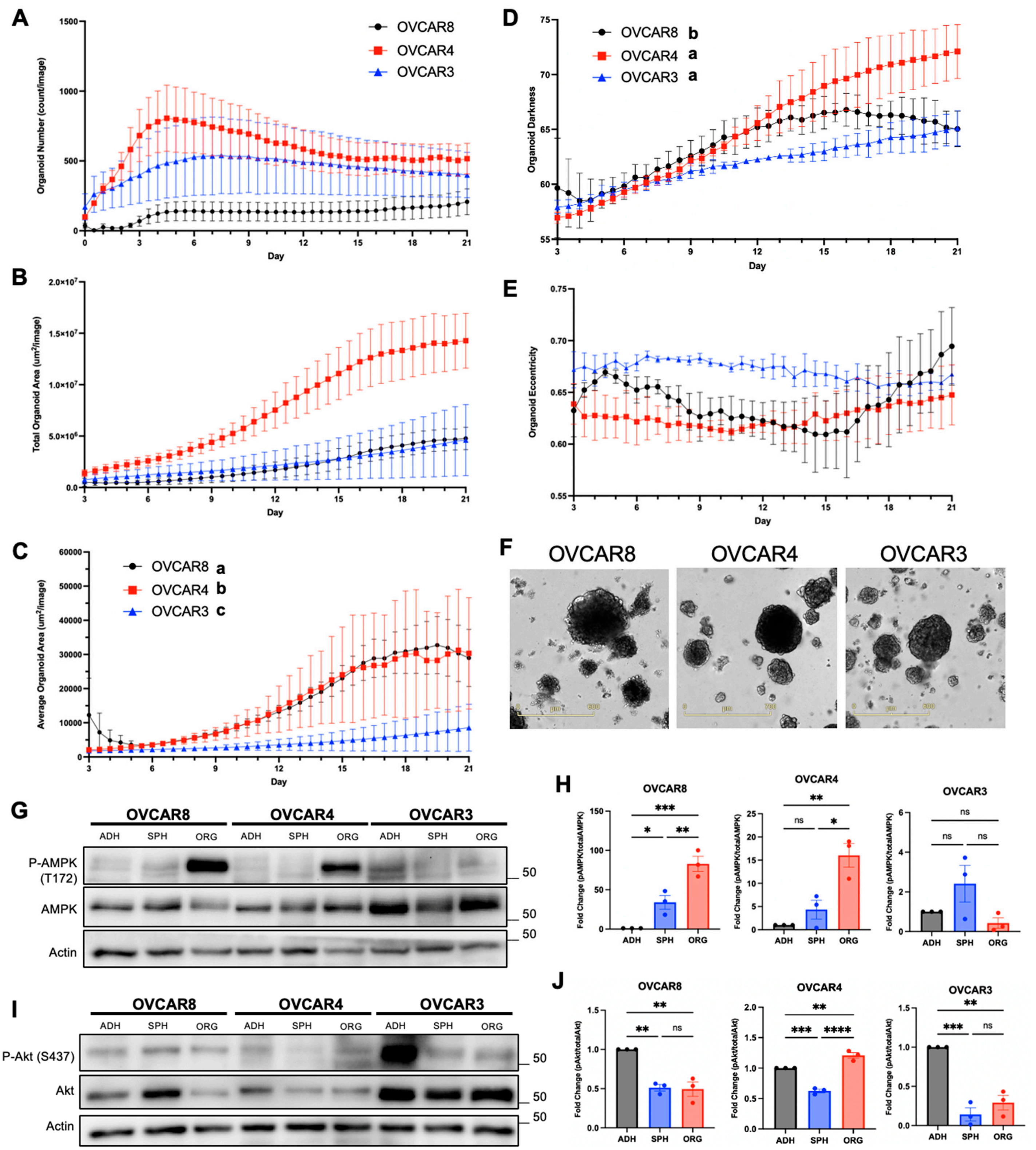

Supplementary Figure S3. Spheroid to organoid comparisons in established OVCAR cell lines. (**A,B**) The Incucyte® S3 software automatically identified each organoid and outlined its border to measure organoid number and total organoid area from each image or well. (**C**) This data was used to calculate the average organoid area (total organoid area / organoid number) over time to demonstrate the growth of individual organoids within each cell line. (**D,E**) The Organoid Module

measured organoid darkness to observe the density of cells or debris within a structure and organoid eccentricity to observe the difference in shape. (**F**) Representative brightfield images of OVCAR8, OVCAR4, and OVCAR3 organoids. Scale bar is 600µm. (**G,H**) Relative protein expression and quantified fold change of phosphorylated AMPK at T172 (P-AMPK) to total AMPK in adherent (ADH), spheroid (SPH) and organoid (ORG) cultures. (**I,J**) Relative protein expression and quantified fold change of phosphorylated Akt at S437 (P-Akt) to total Akt in adherent (ADH), spheroid (SPH) and organoid (ORG) cultures. Graphs were generated using GraphPad Prism 10 demonstrating mean±SEM. Statistical analysis for C was completed on the simple linear regression curve of each cell line over all timepoints and for D,E was completed on only Day 21 data both using an ordinary one-way ANOVA with Tukey's multiple comparisons test and displayed with compact lettering for each cell line indicated as bold letters; for H and J was a Student's t test for each cell line (n=3, *p<0.05, **<p0.01, ***p<0.001, ****p<0.0001).

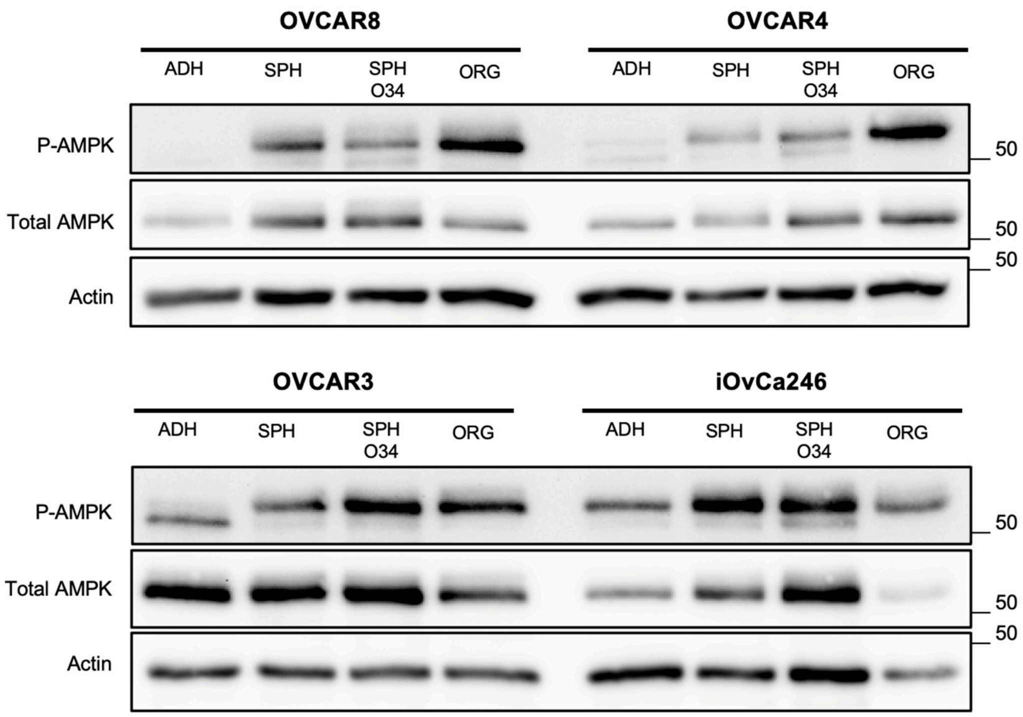

Supplementary Figure S4. The AMPK protein activity is not affected by the supplements added in EOC organoid specific media. Relative protein expression of phosphorylated AMPK at T172 (P-AMPK) to total AMPK in adherent (ADH), spheroid (SPH), spheroids grown in EOC organoid specific media (SPH O34) and organoids (ORG). Spheroids were cultured for 3 days under each media condition.

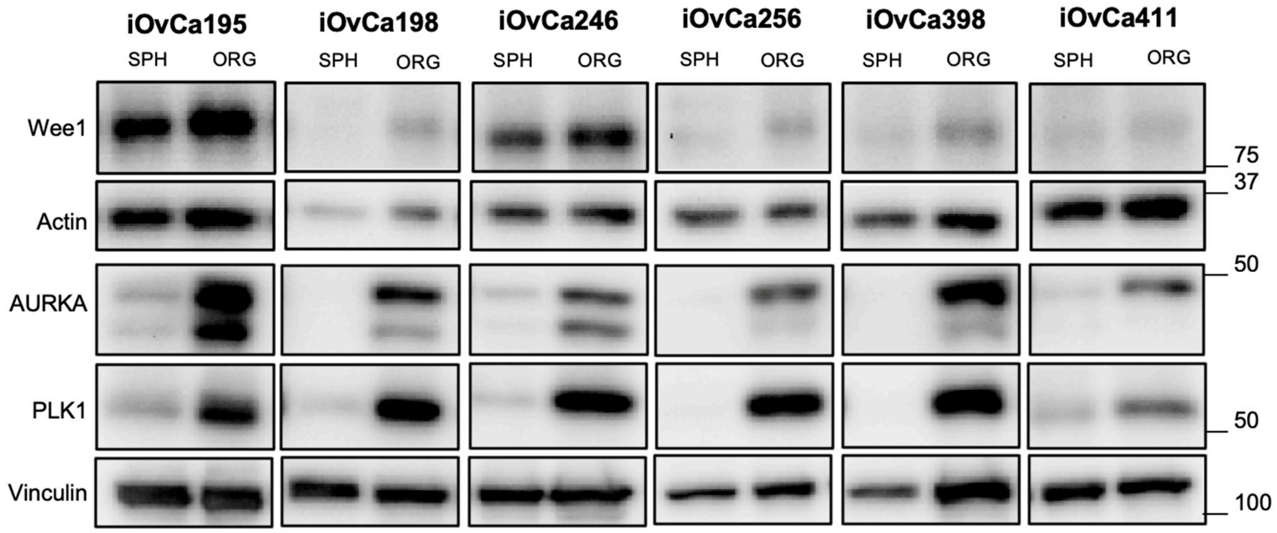

Supplementary Figure S5. Key proteins in the regulation of the G2/M checkpoint pathway. Relative protein expression of Mitosis inhibitor protein kinase Wee1 (Wee1), aurora kinase A (AURKA), and polo-like kinase 1 (PLK1) in spheroids (SPH) and organoids (ORG).

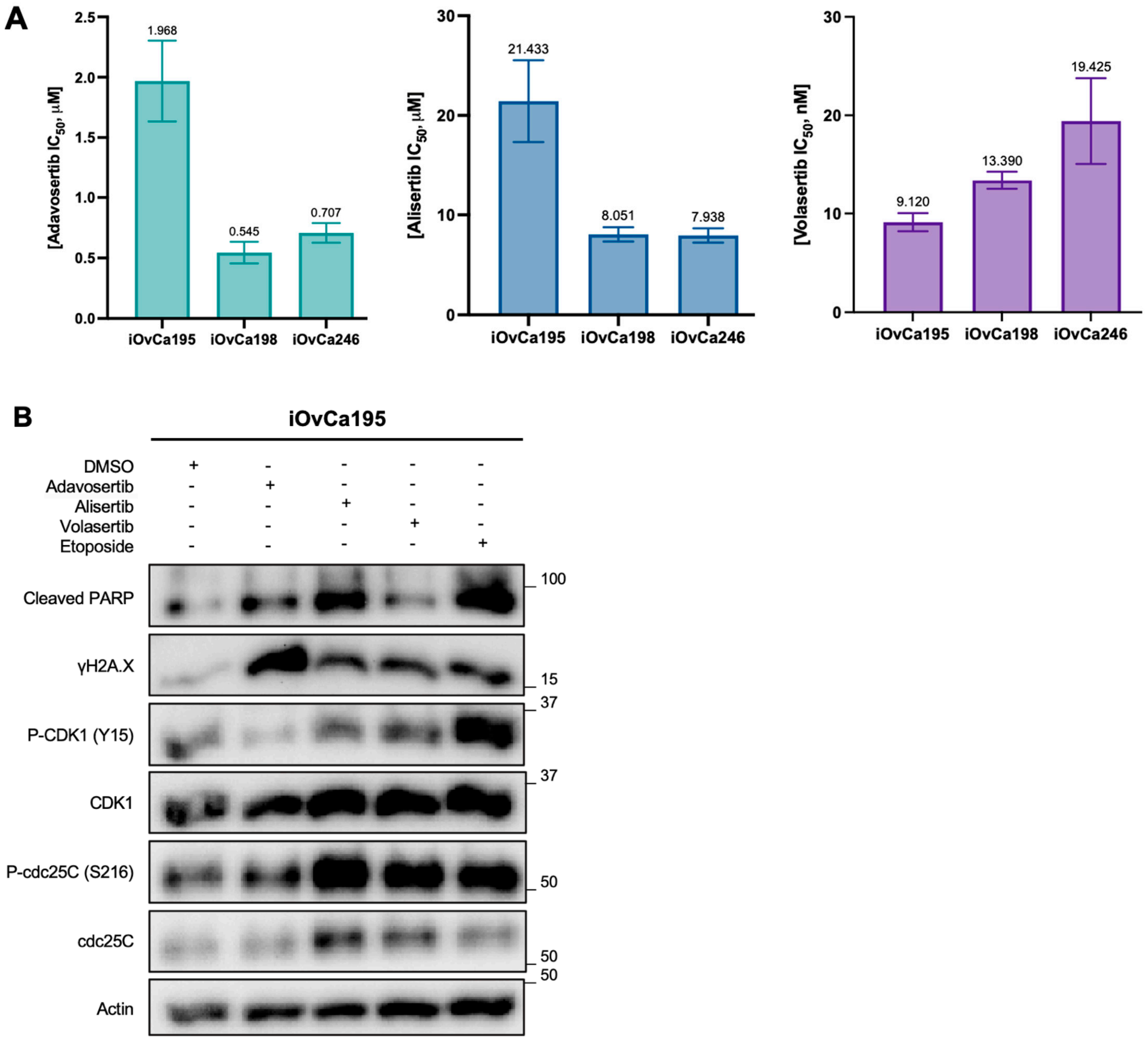

Supplementary Figure S6. G2M checkpoint inhibitor activity levels in cells. (**A**) Bar graphs demonstrating the differing Adavosertib, Alisertib and Volasertib IC50 values in adherent culture conditions for each cell line with the means above each bar. (**B**) Relative protein expression of P-CDK1, total CDK1, cdc25C, γH2A.X and cleaved PARP with 24-hour treatments of DMSO, Adavosertib (700nM), Alisertib (8uM), Volasertib (9nM) and etoposide (10uM). Graphs were generated using GraphPad Prism 10 and presented in mean±SEM.

Supplementary Table S1. EOC organoid specific media formula.

Additive	Company	Catalogue #	[Stock]	[Final]	100mL
Advanced DMEM/F-12	Invitrogen	12634010			95.23 mL
B-27™	Invitrogen	17504044	50x	1x	2 mL
GlutaMAX™	Invitrogen	35050061	200 mM	1x	1 mL
HEPES	Wisent	330-050-EL	1 M	10 mM	1 mL
N-Acetyi-L-cysteine	MilliporeSigma	A9165-25G	500 mM	1.25 mM	250 uL
Recombinant Human Noggin	R&D Systems	6057-NG-100	100 ug/ml	100 ng/mL	100 uL
Nicotinamide	MilliporeSigma	N0636-500G	1 M	1 mM	100 uL
Human FGF-10	Peprotech Inc.	100-26-100UG	100 ug/ml	100 ng/mL	100 uL
Y27632	MilliporeSigma	M1817-10MG	10 mM	10 uM	100 uL
Forskolin	MilliporeSigma	1099/10	10 mM	10 uM	100 uL
Human EGF	Peprotech Inc.	AF-100-15-100UG	100 ug/ml	20 ng/mL	20 uL

Supplementary Table S2. Primer sets for RT-qPCR validation of RNA-sequencing results.

Gene	Forward Primer	Reverse Primer	Product Length
AURKB	CTGTCACCCCATCTGCACTT	TGTGAAGTGCCGCGTTAAGA	122
HMGCR	GACGTGAACCTATGCTGGTCAG	GGTATCTGTTTCAGCCACTAAGG	119
IQGAP3	GTTCGGCAGAAGTTTGCTGAGC	CACTCCAGGTAAATCTTCCGCTG	113
SQLE	CTCCAAGTTCAGGAAAAGCCTGG	GAGAACTGGACTCGGGTTAGCT	136
GAPDH	CATGAGAAGTATGACAACAGCCT	AGTCCTTCCACGATACCAAAGT	113

Supplementary Table S3. Summary of Results.

Cell Line	Spheroids		Organoids		Incucyte© Organoid Analysis		
	Size	Morphology	Size	Morphology	Darkness	Eccentricity	Shape
OVCAR8	Large	Compact	Large	Dense	65.0 ± 0.9	0.695± 0.038	Irregular
OVCAR4	Large	Grape-like	Large	Dense	72.1 ± 1.4	0.648 ± 0.017	Round
OVCAR3	Large	Grape-like	Large	Dense	65.1 ± 0.9	0.667 ± 0.009	Irregular
iOvCa195	Small	Sparse	Medium	Complex	61.2 ± 0.4	0.590 ± 0.013	Round
iOvCa198	Small	Sparse	Small	Dense	57.8 ± 0.7	0.683 ± 0.003	Irregular
iOvCa246	Medium	Grape-like	Small	Dense	59.9 ± 1.2	0.654 ± 0.014	Round
iOvCa256	Medium	Grape-like	Medium	Complex	55.6 ± 0.2	0.651 ± 0.008	Round
iOvCa398	Large	Compact	Small	Complex	58.2 ± 0.3	0.714 ± 0.008	Irregular
iOvCa411	Medium	Sparse	Small	Dense	57.5 ± 2.1	0.685 ± 0.014	Irregular
